# Classification of Hot-Rolled Plates Using the Mahalanobis Distance of NMIs in Ti-Stabilized Austenitic Stainless-Steel Produced by Secondary Metallurgy

**DOI:** 10.3390/ma15020684

**Published:** 2022-01-17

**Authors:** Franci Vode, Franc Tehovnik, Gorazd Kosec, Darja Steiner Petrovič

**Affiliations:** 1 Institute of Metals and Technology, Lepi pot 11, 1000 Ljubljana, Slovenia; 2 SIJ-Acroni d.o.o., Cesta Borisa Kidriča 44, 4270 Jesenice, Slovenia; gorazd.kosec@acroni.si

**Keywords:** stainless steel, ferrotitanium, impurities, oxygen, nonmetallic inclusions, scrap recycling, automated inclusion analysis, Mahalanobis distance

## Abstract

Three charges of scrap-based, Ti-stabilized, Cr-Ni-Mo austenitic stainless steel in the form of hot-rolled steel plates were characterized. Based on automated metallographic analyses of representative microstructures, a quality characterization in terms of cleanliness of the hot-rolled steel plates was performed. Elevated contents of impurities, especially Pb, Bi, and oxygen, which affect the hot workability of stainless steels, were detected. The recycled FeTi-cored wire was the main source of the elevated levels of impurities detected in the hot-rolled, Ti-stabilized, stainless-steel plates. Related to this, elevated levels of nonmetallic inclusions (NMIs) and segregations were formed. The three charges were classified based on calculations of the Mahalanobis distance (MD) between the inclusions. The charge with the smallest number of nonmetallic inclusions was set as the reference class. The selection of outlier inclusions based on their MDs and their back-representation into ternary diagrams gave relevant metallurgical information about the abnormalities. The advantage of this technique is that the calculations of the MD and the threshold can be fully automated.

## 1. Introduction

The energy-intensive steel industry is responsible for approximately 25% of the global direct greenhouse-gas emissions [1,2]. Therefore, the key areas of the current research in process metallurgy relate to the circular economy (CE) and a reduction in carbon dioxide emissions [1,2,3,4,5]. CE as a concept of sustainable competitiveness involves the valorization of steelmaking leftovers for internal reuse (i.e., dusts, slags, sludge), enhanced steel scrap recycling, the use of secondary carbon carriers from nonsteel sectors as a reducing agent, and energy sources in the steelmaking process chain, as well as various business models with comprehensive supply chain analyses [3].

The production of new steel by recycling old steel requires up to 10 times less energy than the primary production of steel from iron ore [5]. As foreseen in the steel industry, electric arc furnace (EAF) steelmaking, either scrap-based or based on hydrogen-direct-reduced iron, will contribute substantially to the reduction of CO_2_ emissions [4]. However, there will still be a need to introduce carbon into the EAF process, either to carburize the steel or to create foaming slag to improve the energy efficiency of the steelmaking process. To develop a fully green steel using EAF, it will be necessary to use alternative carbon sources that are either renewable or circular (e.g., biomass, plastic, rubber wastes, etc.) [4].

Production starts with scrap remelting in EAF followed by secondary metallurgy [5]. For a wide variety of steel grades, intensive scrap-steel recycling in an EAF is already common practice [6,7,8,9,10,11,12]. Scrap is usually well sorted already by suppliers and typically has a low impurity content. On the other hand, when end-of-life (EOL) steel scrap is recycled, the different steel grades are generally separated according to main alloying elements (Cr, Ni, Mo). Some nonferrous and nonmetallic contaminants (copper wires, aluminium, plastic from the shredding of cars, concrete residuals from demolition of buildings, etc.) are generally mixed in small quantities with the steel because of the imperfect separation of different materials prior to melting. Some impurities are removed as slag when the mixture is melted or during subsequent refining steps, and some elements evaporate, but some metallic elements cannot be simply removed (copper, lead). Consequently, the exploitation of steel scrap can lead to an overall increase in the impurity concentrations in steels that cannot be removed by metallurgical processes. Many problems are related to excessive levels of impurities that are prone to precipitation, segregation, and/or the formation of various complex nonmetallic inclusions [5,6,7,8,9,10,11,12]. Accordingly, studies that address open issues in scrap recycling are of great importance.

In modern steelmaking, the formation, characterization and control of nonmetallic inclusions are becoming increasingly important, and tailoring inclusions to improve the properties and performance of steels, known as inclusions engineering, is indispensable [13,14,15,16,17,18]. When cleaner steel is required, additional analysis methodologies and techniques are demanded. Metallographic and chemical analyses can be considered direct methods, while total oxygen content is used as an indirect measurement of inclusions to characterize steel’s cleanliness in terms of oxide inclusions and assess the steelmaking process to control the steel’s quality. Automated inclusion analyses are able to overcome several weaknesses of traditional measures [14]. Therefore, a combination of techniques is recommended [16,17,18].

Austenitic Cr-Ni-Mo stainless steels, which contain Mo to enhance pitting corrosion resistance, are used in the chemical and petrochemical industries; the food, pharmaceutical, and textile industries; building components; welding applications; and for tubes, boilers, vehicle tanks, etc. [19]. Modern steelmaking technologies have enabled the more economical removal of carbon from the steel, primarily through the development of argon oxygen decarburization (AOD) and vacuum oxygen decarburization (VOD). Both production methods lead to carbon levels below 0.03 wt. % [20]. Grades with low carbon content are preferred for applications involving uses at elevated temperatures (such as welding), because chromium carbide precipitation is decreased or prevented and resistance to intergranular corrosion is increased [19]. Austenitic stainless steels can be stabilized using titanium to bind interstitial elements such as carbon and nitrogen to prevent the formation of chromium carbides and nitrides [21,22]. Titanium reduces intergranular corrosion and improves the weldability of corrosion-resistant Cr-Ni steels [22,23]. The stabilized austenitic grades require a titanium addition of at least 5 times the carbon content or 4–6 times the sum of the carbon and nitrogen contents of the steel [24].

Initially, the role of the titanium added to steel as ferrotitanium was mainly to reduce the grain size and to act as a deoxidizer [25]. Titanium dissolved in steel is characterized by a high affinity for oxygen. Titanium lowers the activity of the oxygen in iron [22]. Since surface defects can occur on final steel products, control of the formation of oxide inclusions in Ti-bearing stainless steels is necessary. In addition, the deoxidation products can potentially form deposits within a submerged entry nozzle and thus clog the nozzle [26].

In steels, titanium is also highly reactive with carbon, nitrogen, and sulphur [27]. Nitrogen is an element that causes a great deal of concern. Since titanium nitride forms in preference to titanium carbide, based on thermodynamic considerations, it is essential (i) to add sufficient titanium to chemically bind the nitrogen first and then the carbon or (ii) to reduce the nitrogen as much as possible using other steelmaking techniques [27].

Titanium alloying can be made in the form of metal scrap, sponge, or as a ferrotitanium alloy. Ferrotitanium addition for binding interstitial elements is usually performed after the steel is refined in a ladle furnace (LF) [26]. For the cored wire, FeTi alloying is made as deep and late into the ladle as possible. Prior to the alloying, the liquid steel should be thoroughly deoxidized to reduce the oxidation of, and thus maximize the recovery of, the titanium [27]. In the case of titanium-stabilized stainless steels, the LF refining process involves deoxidation by Al followed by the addition of the Ti-alloy [26,28,29].

The quality requirements for FeTi wire are chemical composition, size, and cleanliness [21]. The levels of tolerable elements vary depending on the application. Usually, the Ti-Al-V (titanium, aluminium, vanadium) tolerances are defined. In addition, the maximum content of nitrogen, lead, bismuth, and tin are very important [27]. The phase composition of ferrotitanium depends on the content of Ti and impurities. The nature of the impurities that are present can be ascribed to different resources and processing routes [22,23].

With the increasing use of titanium alloys in various industries, e.g., in aerospace, shipbuilding, etc., large volumes of EOL titanium scrap are available and find a way as Ti-containing cored wires. Therefore, FeTi cleanliness has become an issue [27,30,31], and there is a strong demand for high-quality FeTi. Recently, a novel methodology was proposed for preparing low-oxygen, high-titanium ferroalloy from high-titanium slag and iron concentrate with a multistage and deep reduction procedure [32]. Moreover, the sustainable recycling of titanium scraps, along with a high-purity titanium production route via molten-salt electrolysis, was also proposed to separate the impurity elements and the titanium [33].

The aim of this study was to evaluate the influence of impurities on nonmetallic inclusion formation and the quality of hot-rolled plates manufactured from titanium-stabilized, austenitic stainless steels of AISI316Ti grade, i.e., from Cr-Ni-Mo steels produced by secondary metallurgy. In particular, the influence of the impurities present in a recycled ferrotitanium and the detected oxygen contents in the steel were highlighted. The metallographic results were compared and discussed with reference to the results obtained using mathematical modelling. NMI features obtained by automated metallographic analyses were employed to numerically estimate the quality of the hot-rolled plates. In the relevant scientific literature, NMIs are classified according to their type (carbides, nitrides, sulphides, oxides, etc.), and other, more complex inclusion types is a well-covered topic [6,7,9,10,11,13,14,15,16,17,18]. However, the use of NMI features for the detection of production abnormalities in terms of two-class classification has not been reported.

In the present study, the Mahalanobis distance (MD) was employed as a continuous measurement scale in a multidimensional system/space (NMI features in this case) between two classes of data [34,35]. The first class is used as a reference or normal class, and the second class (frequently abnormal) is compared against the first. The calculation of the MD is numerical and, from the user’s point of view, undemanding and is therefore appropriate for automation [34,35].

The novelty of the study lay in the selection of inclusions based on their MDs outlying from the reference class. Inclusions classified as outliers are back-represented in ternary diagrams, thus giving relevant metallurgical information. The results were derived from a research collaboration between industry and a laboratory characterization of materials.

## 2. Methods and Materials

### 2.1. Sampling

For the chemical analyses, the steel samples were selected randomly.

For the metallographic analyses, the representative steel samples were selected using sampling in the longitudinal (i.e., rolling) direction. Steel samples taken from the hot-rolled plates are designated as Sample A, Sample B, and a reference Sample R.

Samples of recycled FeTi-cored wire were selected randomly.

### 2.2. Chemical Analyses

Chemical compositions of the materials under investigation were determined by an XRF analyser for the chemical analysis using a Thermo Scientific Niton XL3t GOLDD+ instrument (Niton Europe GmbH, Munich, Germany) and an optical emission spectrometer with inductively coupled plasma (ICP-OES), an Agilent 720 instrument (Agilent Technologies, Inc., Santa Clara, CA, USA).

The ICP-OES detection limits for Pb and Bi were greater than or equal to 0.01 wt. %. Combustion methods using ELTRA analysers (ELTRA, GmbH, Haan, Germany), i.e., ELTRA CS-800 for carbon and sulphur and ELTRA ON-900 for oxygen and nitrogen, were also applied.

#### 2.2.1. Steels

The 18 mm-thick hot-rolled plates under investigation were industrially produced from Ti-stabilized, austenitic, Cr-Ni-Mo stainless steels. Their basic chemical compositions are listed in Table 1. In addition, the mechanical properties (i.e., impact toughness and hardness) of the steels were measured. The results are presented in Table 1.

Impact toughness was measured according to the international standard ISO 148-1:2017. The standard specimens for the Charpy V-notch impact testing, of dimensions 10 mm × 10 mm × 55 mm, were used for the measurements performed at 25 °C, −45 °C, and −196 °C. A pendulum impact tester MFL PSW 300 (MFL Prüf- und Meβsysteme GmbH, Mannheim, Germany) was used.

The Vickers hardness (HV10) was measured according to the international standard ISO 6507-1:2018 using an Instron Wilson-Wolpert Tukon 2100B tester (Instron GmbH, Darmstadt, Germany).

#### 2.2.2. Cored Ferrotitanium Wire

The chemical compositions of the recycled ferrotitanium (FeTi) wire used in the manufacturing of the Ti-stabilized, austenitic, Cr-Ni-Mo stainless steels are provided in Table 2.

### 2.3. Metallography

The samples were ground and polished. Optical imaging was performed using an Microphot FXA (Nikon, Tokyo, Japan) optical microscope equipped with an Olympus DP73 camera (Olympus, Hamburg, Germany) and the Stream Motion Programme. Further metallographic analyses were performed using a scanning electron microscope with a ZEISS-Crossbeam 550/EDAX (Carl Zeiss AG, Oberkochen, Germany) instrument for the FE-SEM/BSE/EDS. In addition, a JEOL JSM-6500F (JEOL Ltd., Tokyo, Japan) scanning electron microscope was used for certain FE-SEM/EDS analyses. Where necessary, etching with the aqua regia etchant (10 mL HNO_3_ + 30 mL HCl + 20 mL glycerine) was applied.

#### Automated Analysis of Inclusions

An automated quantitative and qualitative analysis of inclusions was performed by FE-SEM/BSE/FEATURE analyses using the JEOL JSM-6500F (JEOL Ltd., Tokyo, Japan) scanning electron microscope equipped with an Inca Energy 450 EDS system using an x-sight LN2 (Oxford Instruments plc, Abingdon, UK) detector. For each sample, the analysed surface area was 2.0 × 1.0 mm^2^ at a magnification of 2000×. The applied electron beam conditions were a 15 kV accelerating voltage and a 10 mm working distance. Images had a resolution of 1024 × 832 pixels.

In the automated FEATURE analysis, the detection size for the inclusions was set to be greater than or equal to 3 pixels (within the 0.12 µm ECD bin size). This setting was capable of capturing relevant nonmetallic inclusions of the microstructure. According to the setting, FEATURE regarded various elemental segregations (generally of 10 μm^2^ and more) as inclusions.

### 2.4. Composition of Inclusions

The compositions of the inclusions are graphically represented according to the elemental content in wt. % based on data (i.e., measurements using the FE-SEM/EDS/FEATURE technique). MATLAB software was used (MATLAB, version R2016a, MathWorks, Inc., Natick, MA, USA).

### 2.5. Calculation of the Mahalanobis Distance

The calculations of the Mahalanobis distances (MDs) were performed according to [34,35]. The MD is a continuous measurement scale in a multidimensional system/space between only two classes of data, a reference (normal) and observed (abnormal) class. The MD is a vectorial-matrix equation and calculated as the distance between the centre of the normal class and each observation being evaluated. This takes into account the codependency of the data using a covariance matrix.

## 3. Results

### 3.1. Microstructures of the Materials

#### 3.1.1. Hot-Rolled Plates of Ti-Stabilized Cr-Ni-Mo Steel

**Figure 1:**
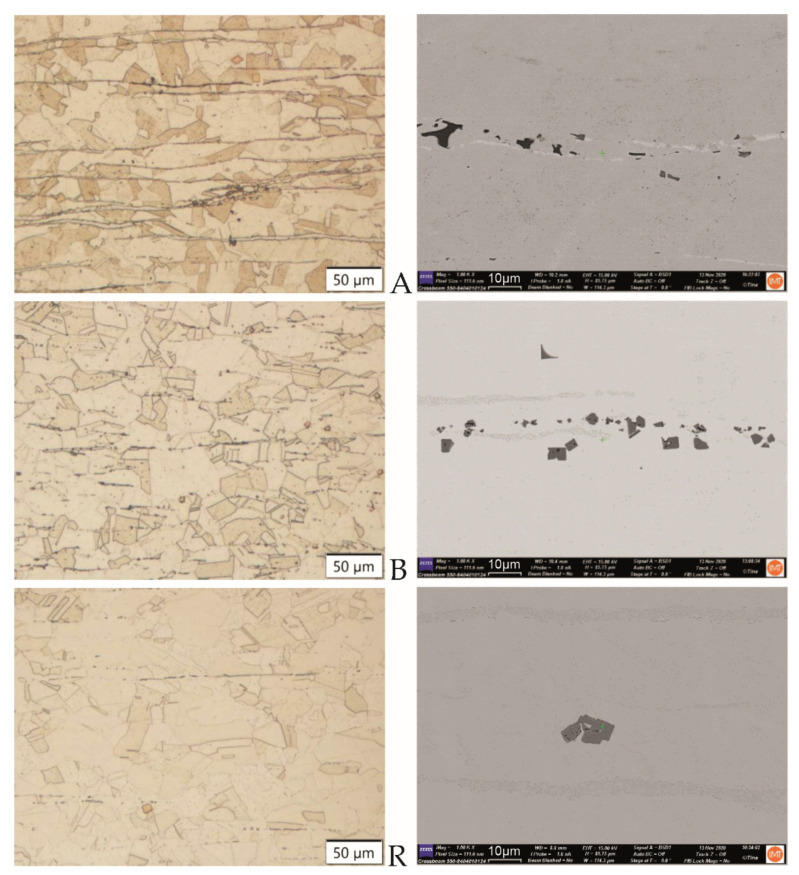
Microstructures of the hot-rolled plates A, B, and R in the rolling direction. (**Left**: OM, mag. 200×, etched. **Right**: FE-SEM-BSI, 1000×, polished).

Typical microstructures of the hot-rolled plates in the rolling direction are shown in Figure 1. In the BSE imaging, heavier elements (higher Z) appear brighter than lighter elements. In the back-scattered electron images, the nitrides are very visible (Figure 1, see micrographs on the right-hand side). The nitrides typically appear with geometric angular shapes. Because Ti is a lighter element than Fe, it is coloured darker than the iron matrix. Segregated elements are seen as darker (because lighter elements are present) or lighter stringers (containing heavier elements than Fe).

#### 3.1.2. Recycled FeTi-Cored Wire

**Figure 2:**
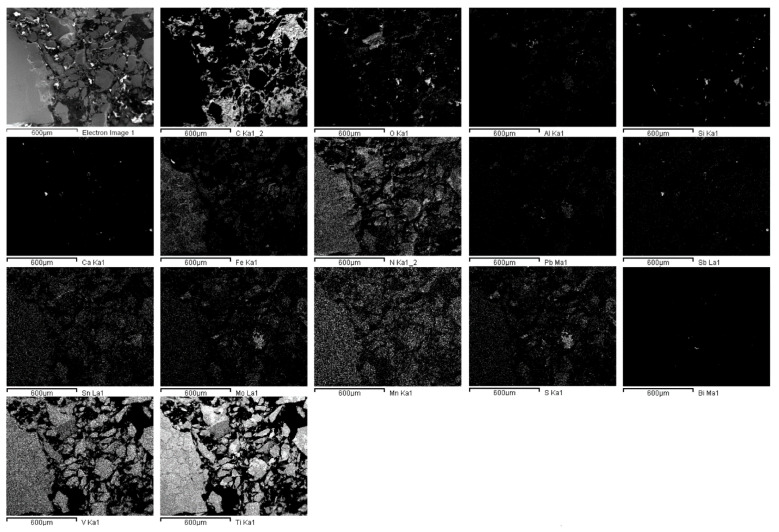
SEM image and the corresponding X-ray elemental mappings showing the distribution of elements in the FeTi-cored wire (C, O, Al, Si, Ca, Fe, N, Pb, Sb, Sn, Mo, Mn, S, Bi, V, Ti).

The recycled FeTi-cored wire that was used in the manufacturing of Ti-stabilized Cr-Ni-Mo steel demonstrated a very inhomogeneous microstructure and distribution of elements. In Figure 2, FE-SEM/EDS mapping of a randomly selected sample is shown. The material’s inhomogeneity was previously confirmed by chemical analyses (Table 2).

### 3.2. Automated Analysis of Inclusions

#### 3.2.1. Quantity, Size, and Composition of Inclusions Based on Data Obtained from Automated Analyses

FE-SEM/EDS/FEATURE analyses of the hot-rolled plates of Ti-stabilized Cr-Ni-Mo steel A and B and a reference sample R were performed on cross-sections of the samples taken from the steel plates’ rolling direction on populations of 15,858, 16,705, and 11,341 features, respectively.

The numbers of detected precipitates per area, which were the same for all the samples, should indicate the cleanliness of the steel.

With reference to this, the steel charge R was evaluated as a reference material in terms of the best cleanliness. The oxygen-rich sample B exhibited the lowest level of cleanliness.

**Figure 3:**
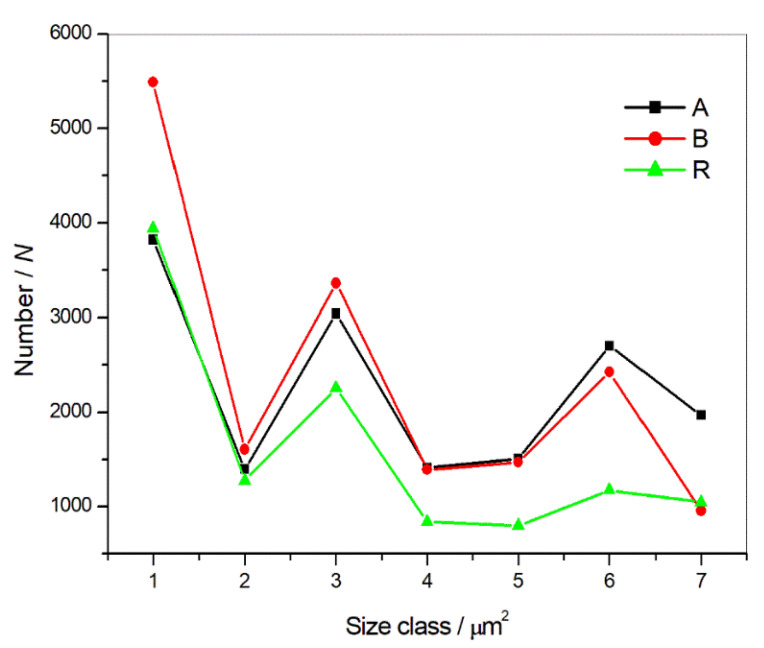
Number and size of inclusions in the hot-rolled steel plates A and B and the reference sample R.

Statistical analyses, the distribution of inclusions (i.e., nonmetallic inclusions), and segregations into size classes showed significant differences between charges (Figure 3, Table 3).

**Figure 4:**
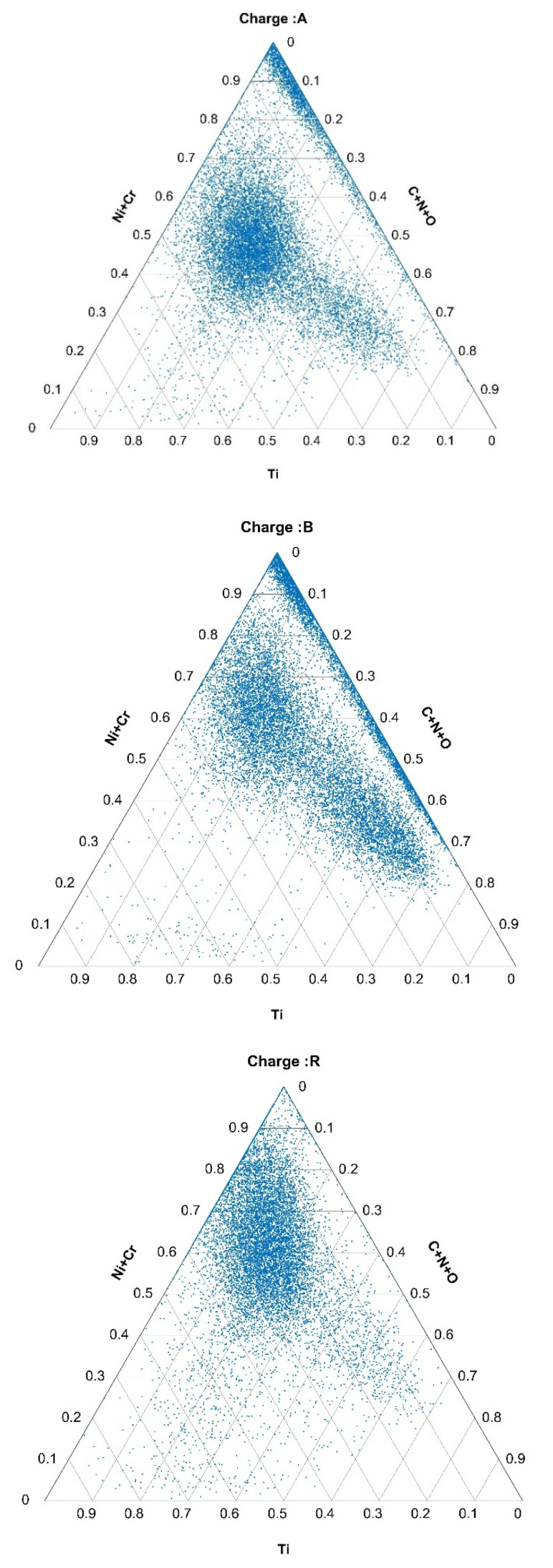
Composition of Ti-, C+N+O-, and Ni+Cr-rich inclusions in the hot-rolled plates of Ti-stabilized Cr-Ni-Mo steel A, B, and R.

**Figure 5:**
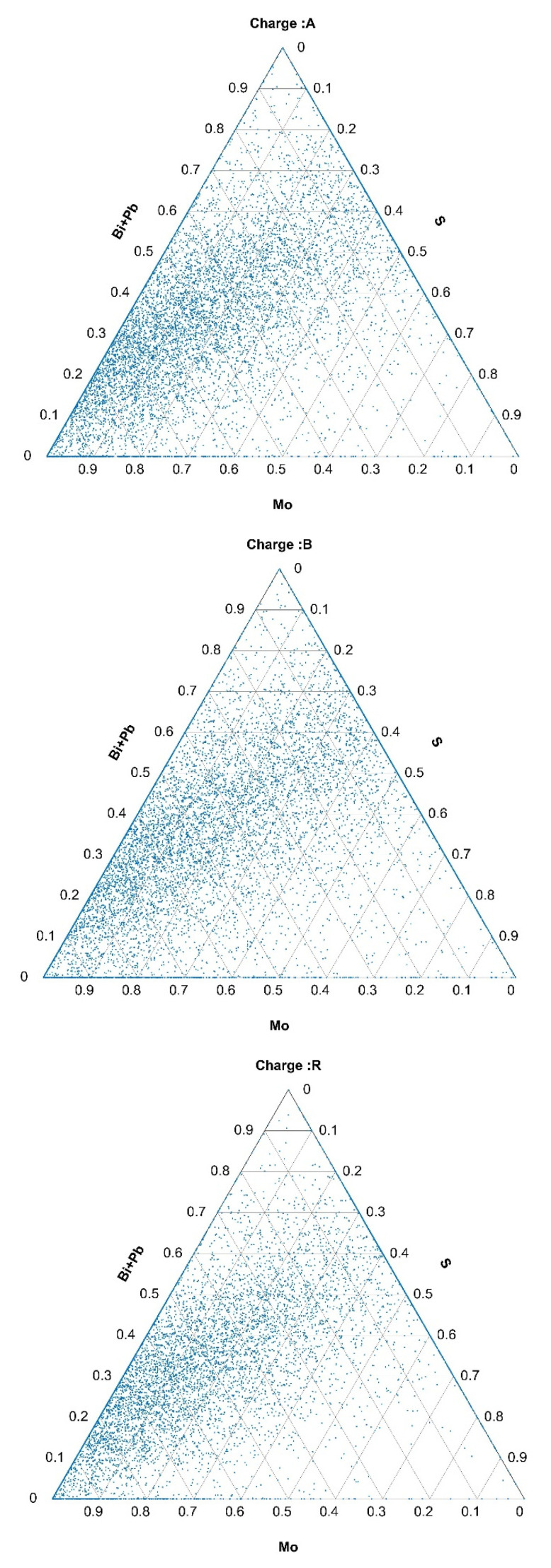
Composition of Mo-, S-, and Bi+Pb-containing inclusions in hot-rolled plates of Ti-stabilized Cr-Ni-Mo steel A, B, and R.

**Figure 6:**
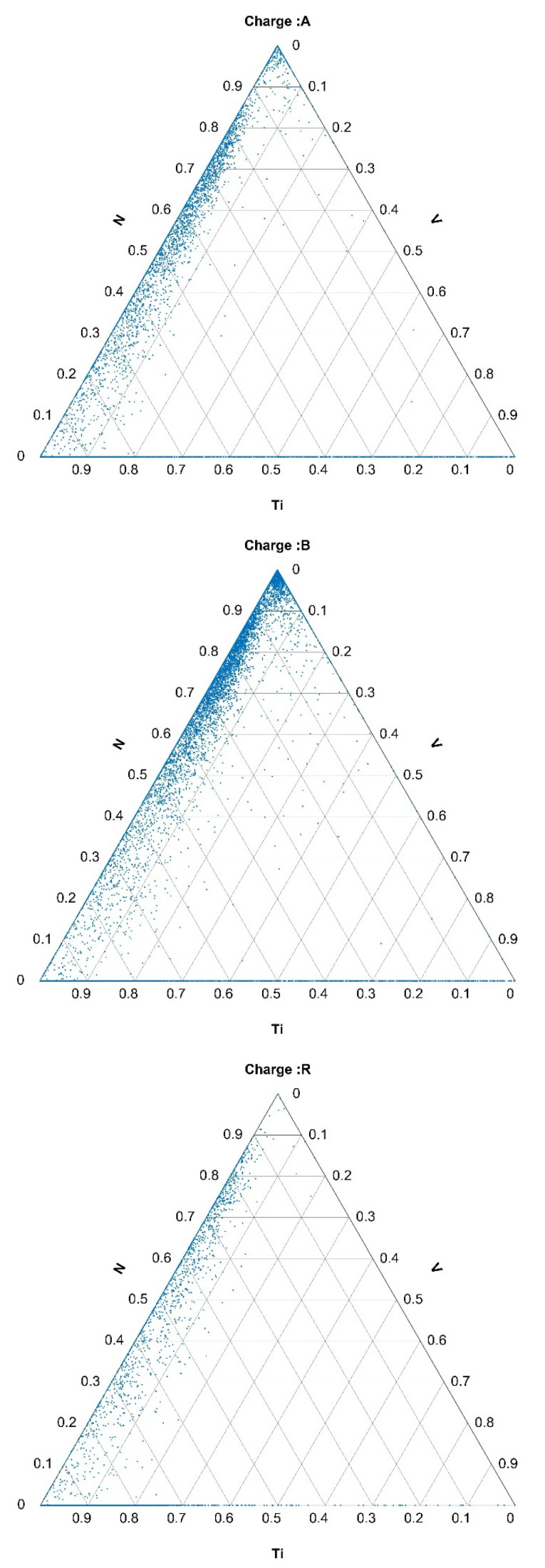
Composition of Ti-, V-, and N-containing inclusions in hot-rolled plates of Ti-stabilized Cr-Ni-Mo steel A, B, and R.

The compositions of the inclusions containing the selected elements are represented in the ternary diagrams in Figure 4, Figure 5 and Figure 6.

#### 3.2.2. Histograms of Calculated Mahalanobis Distances Based on the Data Obtained from the Automated Analysis of Inclusions for Charges A, B, and R

**Figure 7:**
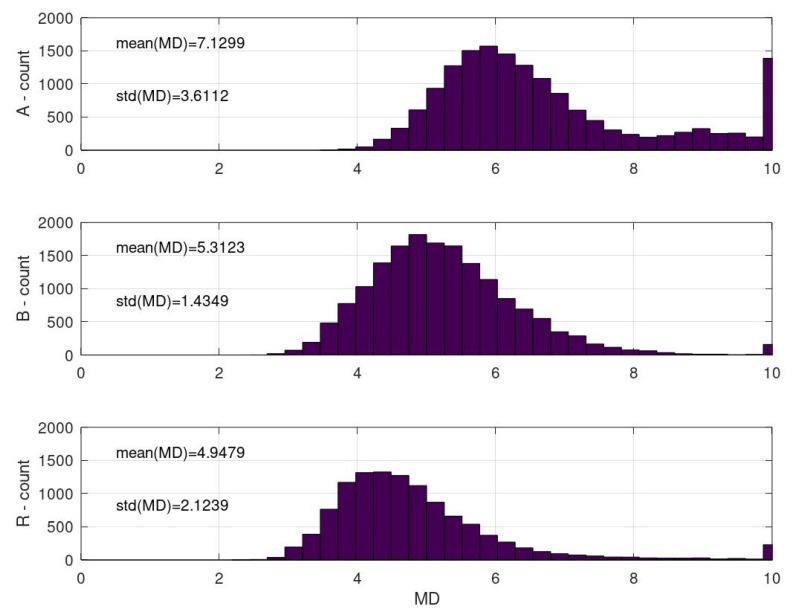
Calculated Mahalanobis distances between inclusions in the hot-rolled steel plates A and B and the reference sample R.

The different mean values of the MDs for charges A, B, and R seemed to be the most obvious change besides the total number of inclusions (Figure 7). Another stand-out difference was the distribution of the MDs in charge A (above approximately 8); the MDs for charges B and R were significantly smaller above that value.

Note that the MD is a unitless measure. A survey of literature data on classification based on the MD suggested four thresholding methods [34,35]. Depending on the requirements and the overlapping of the observed classes, many different options for classifications are possible. In the case of the present study, classes of MDs for charges A, B, and R strongly overlapped, and every two-class separation resulted in a strong population of common inclusions of the two. However, one thresholding method, the probabilistic threshold method, is calculated as:

(1) $$MD_{thresh}=MD_{mean}+\sqrt{\frac{100}{100+\lambda -\omega }}std_{MD}$$

where *MD_mean_* is the mean value and *std_MD_* is the standard deviation of the MD of the reference (normal) class. Lambda and omega are usually parameters of small values. In this case, both were assumed to be equal, thus leading to a value below square root equalling one. Consequently, the threshold was set at $MD_{thresh}=4.9479+2.1239=7.0718\approx 7$.

Note that the threshold value can be easily obtained computationally. However, using human supervision, we preferred to set the threshold at the minimum of the MDA, slightly above 8.

**Figure 8:**
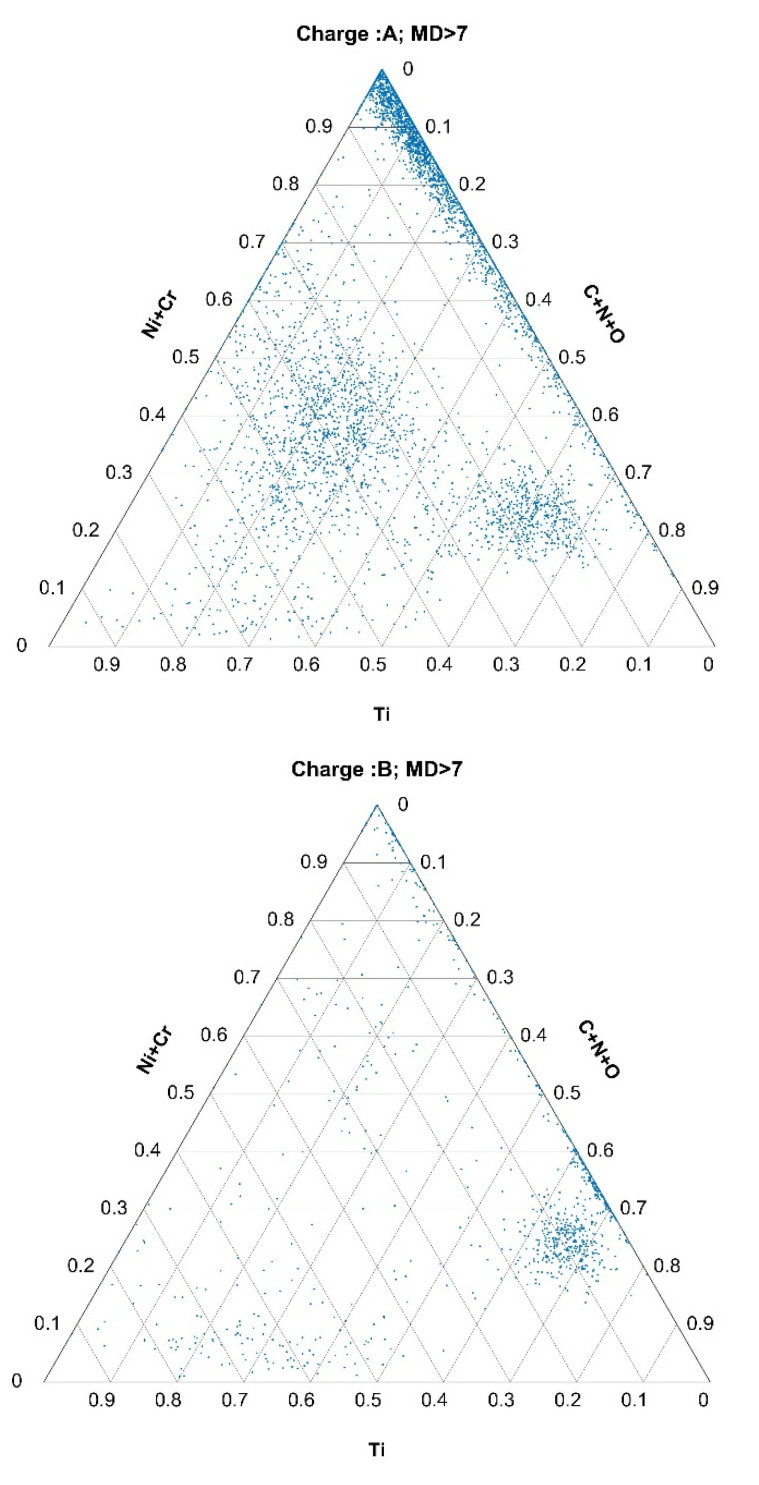

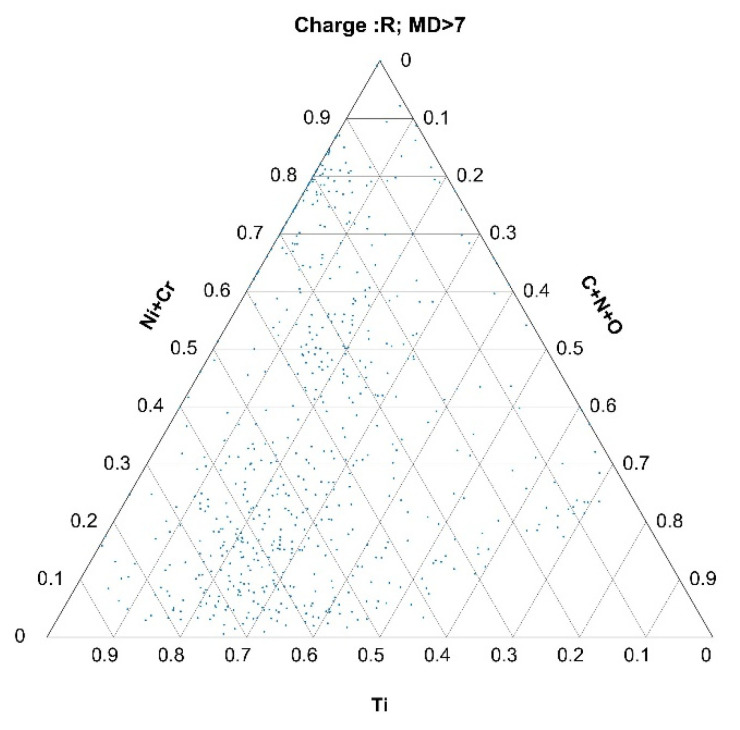
Inclusions classified as outlying (MD > 7) represented in a ternary diagram of Ti, C+N+O, and Ni+Cr for charges A, B, and R.

Applying the obtained $MD_{thresh}$ to all three charges, A, B, and R, ternary diagrams for the system of (Ti)-(C+N+O)-(Ni+Cr) for these nonmetallic inclusions, the thresholds of which were above $MD_{thresh}$, were plotted. Ternary diagrams for these inclusions for charges A, B, and R are shown in Figure 8. Interestingly, these inclusions were mostly bands with very low Ti contents and high contents of Ni+Cr, while the span of C+N+O for these inclusions was between 0 and 0.7.

**Figure A1:**
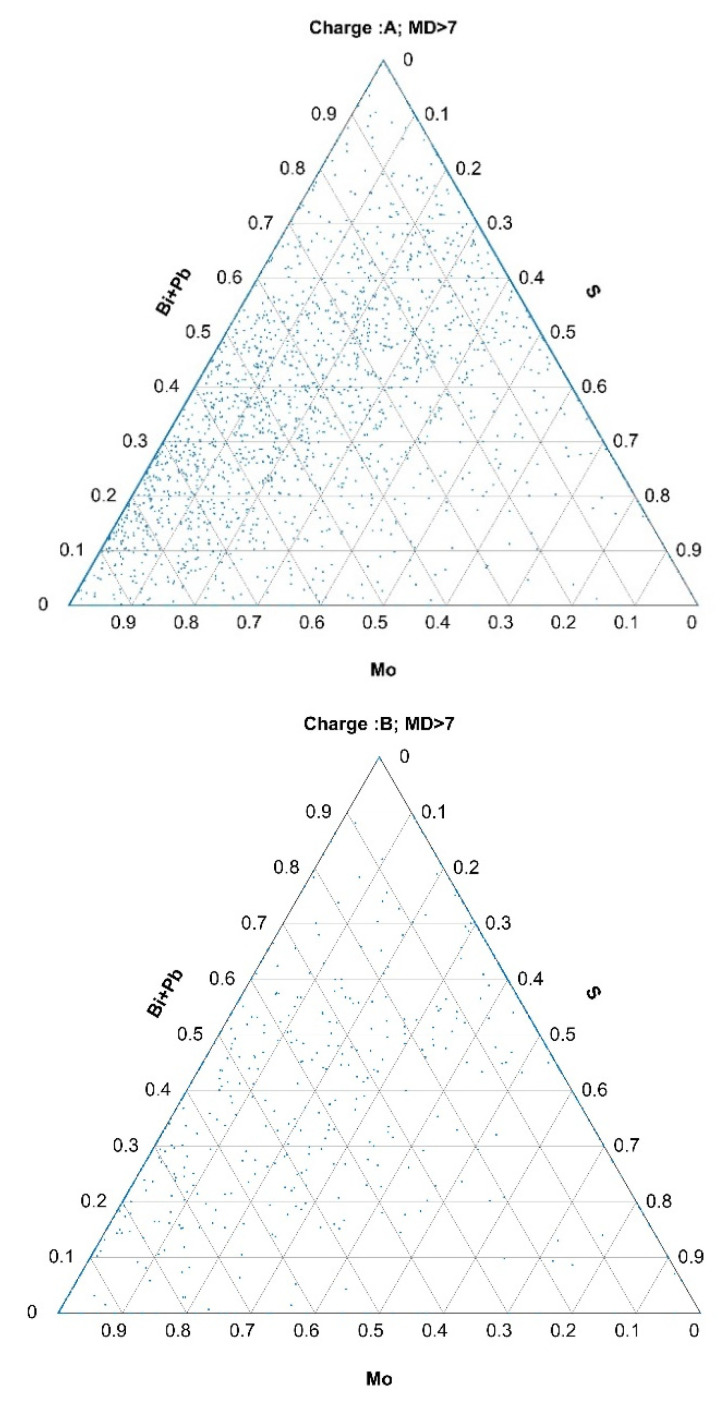

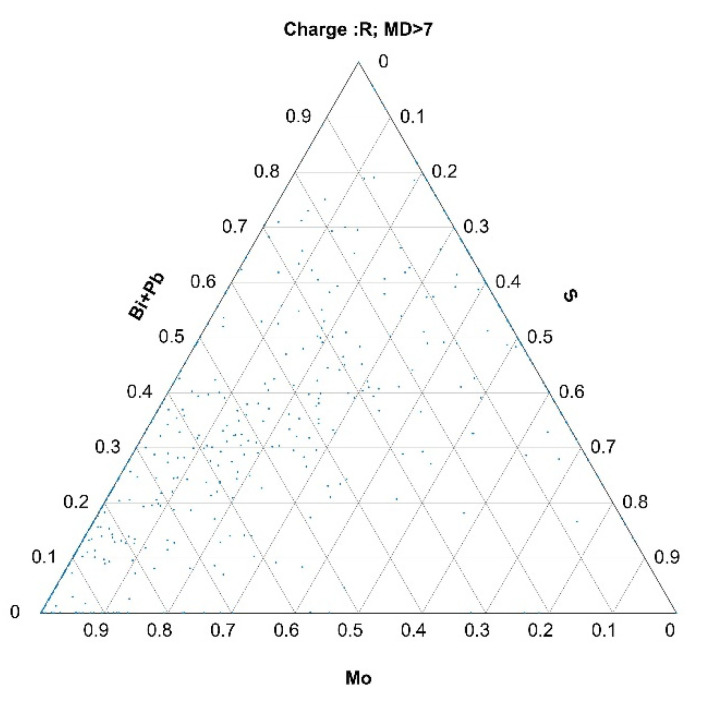
Inclusions classified as outlying (MD > 7) represented in a ternary diagram of Mo, S, and Bi+Pb for charges A, B, and R.

**Figure A2:**
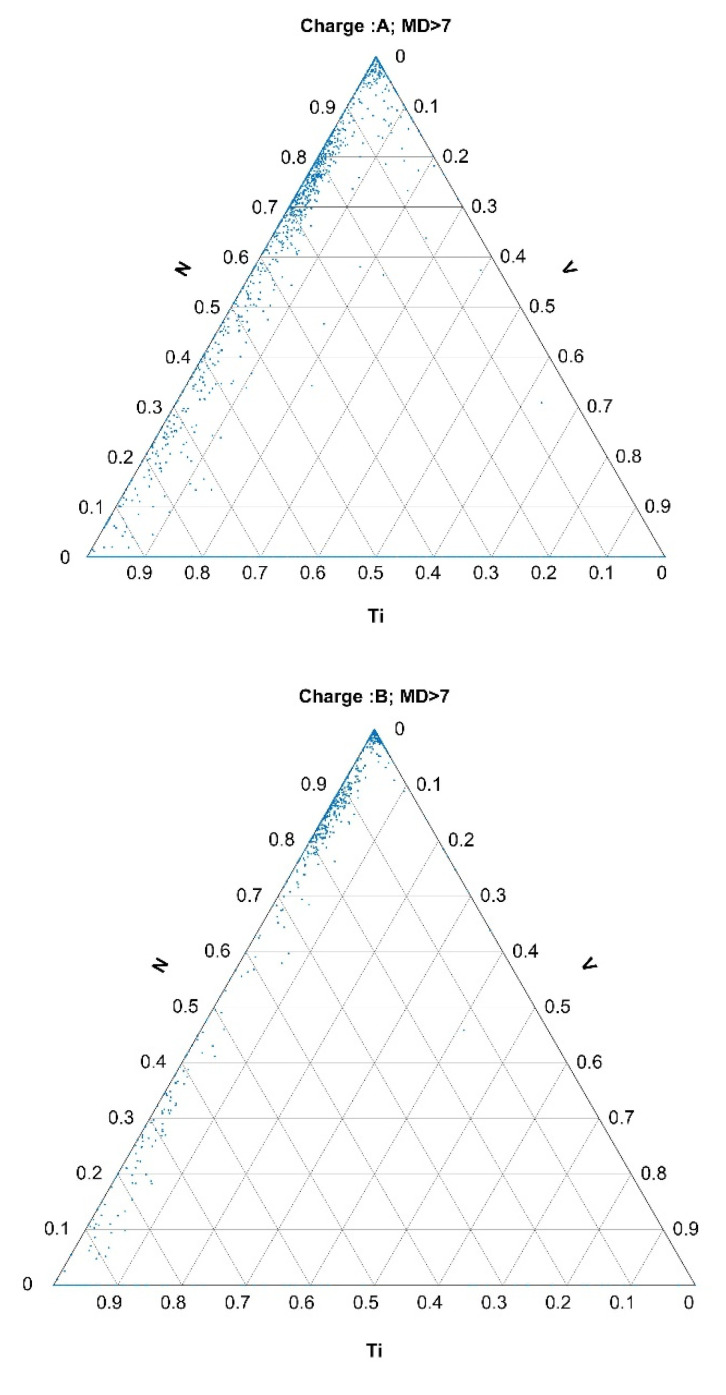

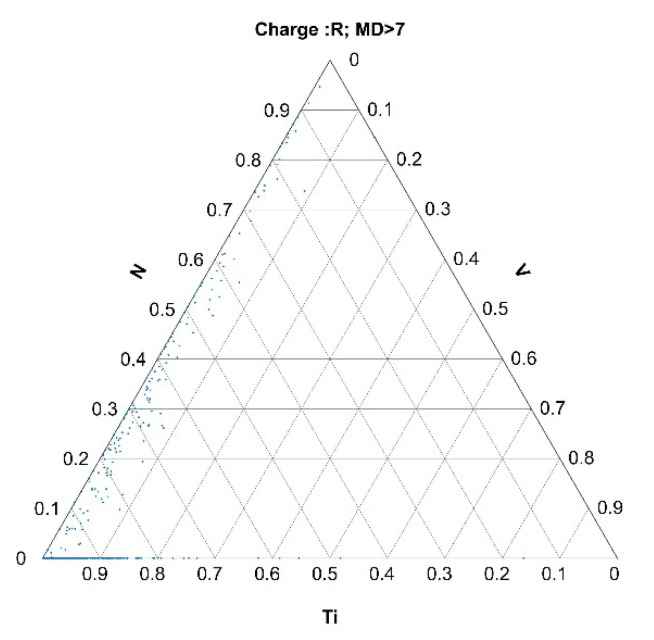
Inclusions classified as outlying (MD > 7) represented in a ternary diagram of Ti, V, and N for charges A, B, and R.

Analogous to these in Figure A1 and Figure A2 are ternary diagrams for the inclusions of Mo, S, and (Bi+Pb) and for the inclusions of Ti, V, N (please see Appendix A). In the case of the inclusions that contained Mo, S, Bi, and Pb, they were present in significantly larger proportions in charges A and B than in the reference charge R. The same was valid for the Ti, V, and N inclusions.

### 3.3. Formation of Nonmetallic Inclusions and Segregations

#### 3.3.1. Nonmetallic Inclusions after FeTi Alloying

**Figure 9:**
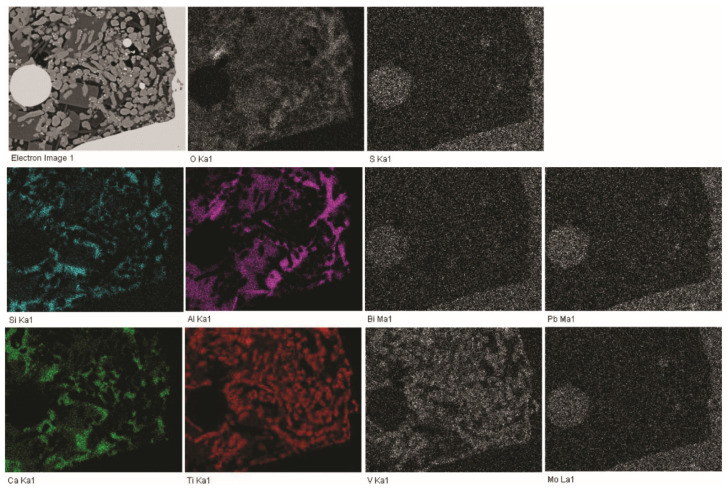
Accumulation of impurities in the steel melt B after FeTi alloying (FE-SEM-EDS; mag. 5000×; SEI and the distributions of elements O, S, Si, Al, Bi, Pb, Ca, Ti, V, Mo).

Figure 9 shows the microstructure of steel B after alloying with FeTi wire. Large accumulations of impurities and oxygen were observed. Moreover, it is obvious that not only titanium but large amounts of additional impurities were introduced into the steel by alloying with a recycled FeTi (see the Ti, O, S, Mo, Bi, and Pb maps in Figure 2).

#### 3.3.2. Nonmetallic Inclusions after FeTi Alloying and Hot Rolling

##### Segregation of Impurity Elements

**Figure 10:**
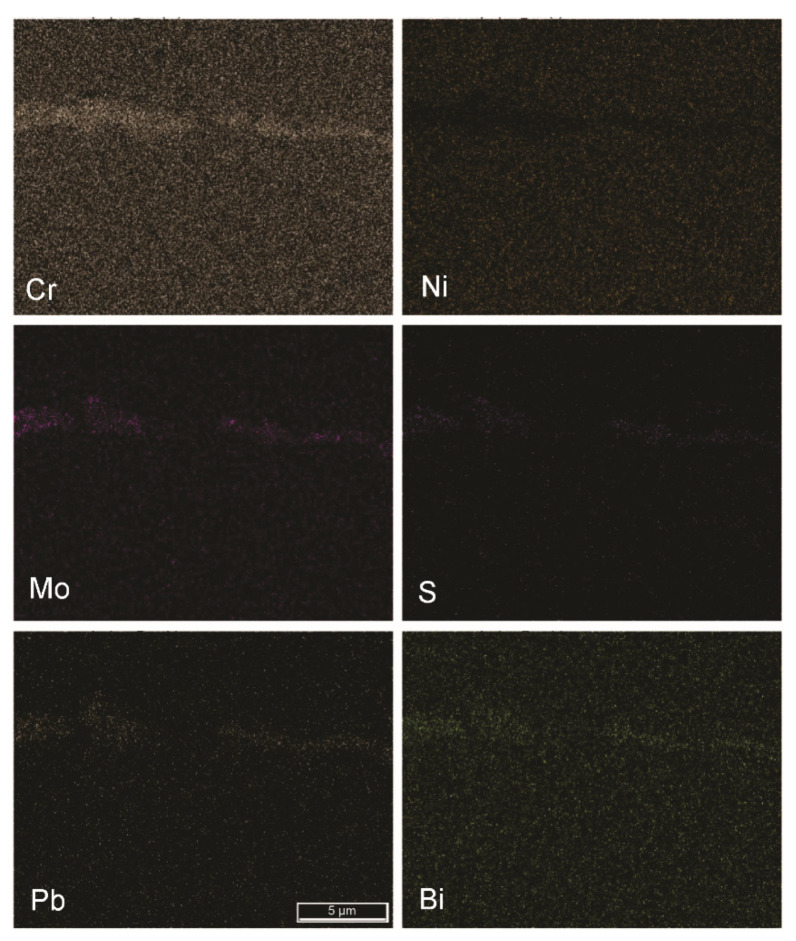
Segregation of Cr, Mo, S, Pb, and Bi in the hot-rolled plate of steel A (FE-SEM-EDS; mag. 5000×).

**Figure 11:**
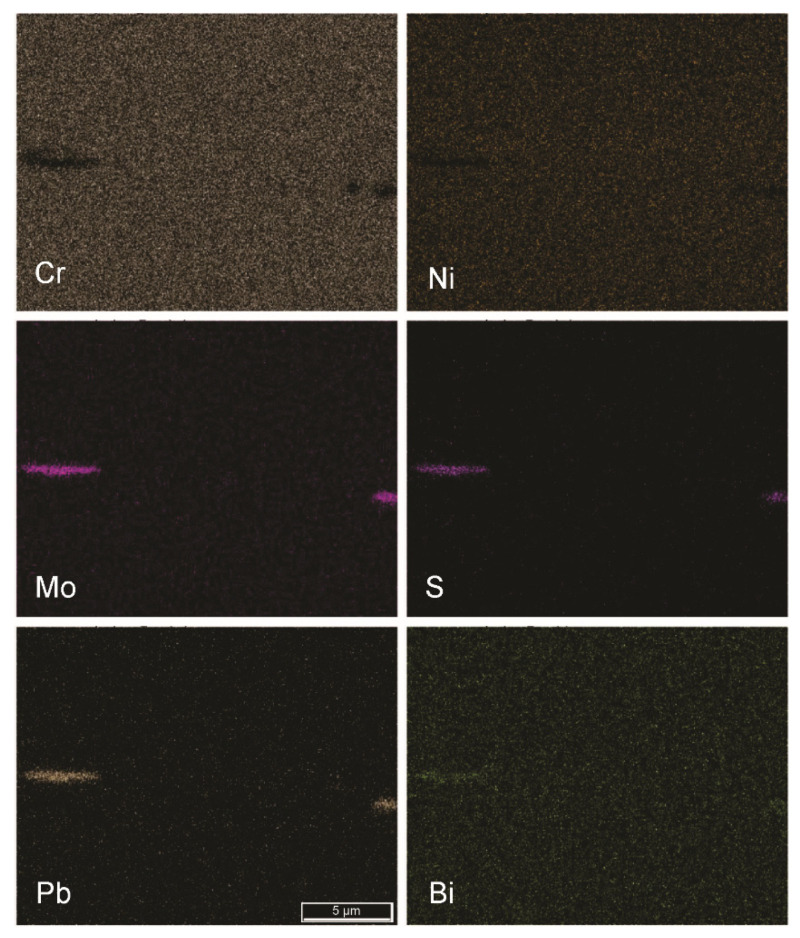
Segregation of Mo, S, Pb, and Bi in the hot-rolled plate of steel B (FE-SEM-EDS mapping; mag. 5000×).

**Figure 12:**
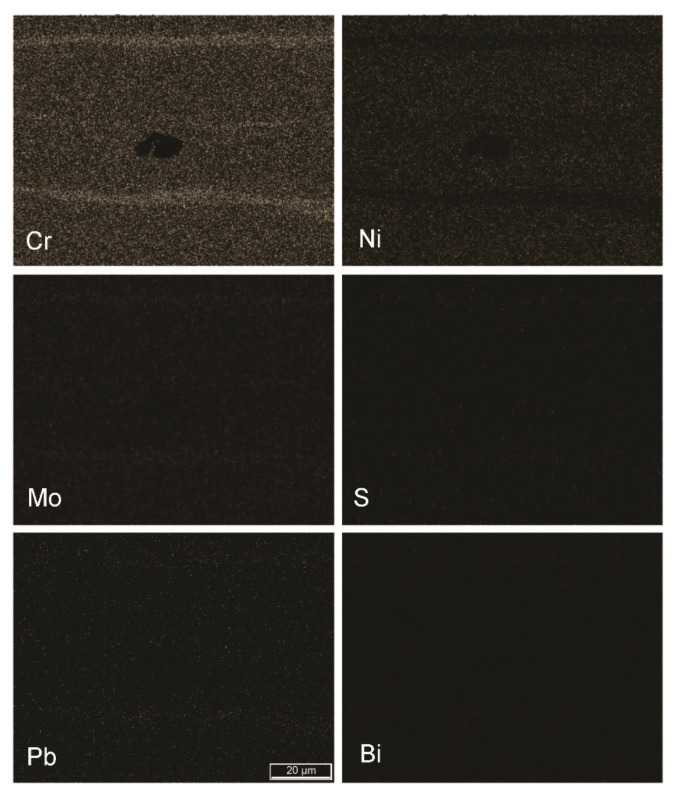
Segregation of Cr, Mo, S, and Pb in the hot-rolled plate of steel R (FE-SEM-EDS mapping; mag. 1000×).

Because of the nature of the chosen automated metallographic analytical technique, elemental segregations were also considered as nonmetallic inclusions. Figure 10, Figure 11 and Figure 12 show the segregations of impurity elements in the analysed steels A, B, and R. In the EDS elemental mappings, Cr segregations were detected, as expected. More unexpected were the results showing the cosegregation of the impurity elements Mo, S, Pb, and Bi. This phenomenon was most pronounced in sample B and was not observed in the reference sample R.

**Figure 13:**
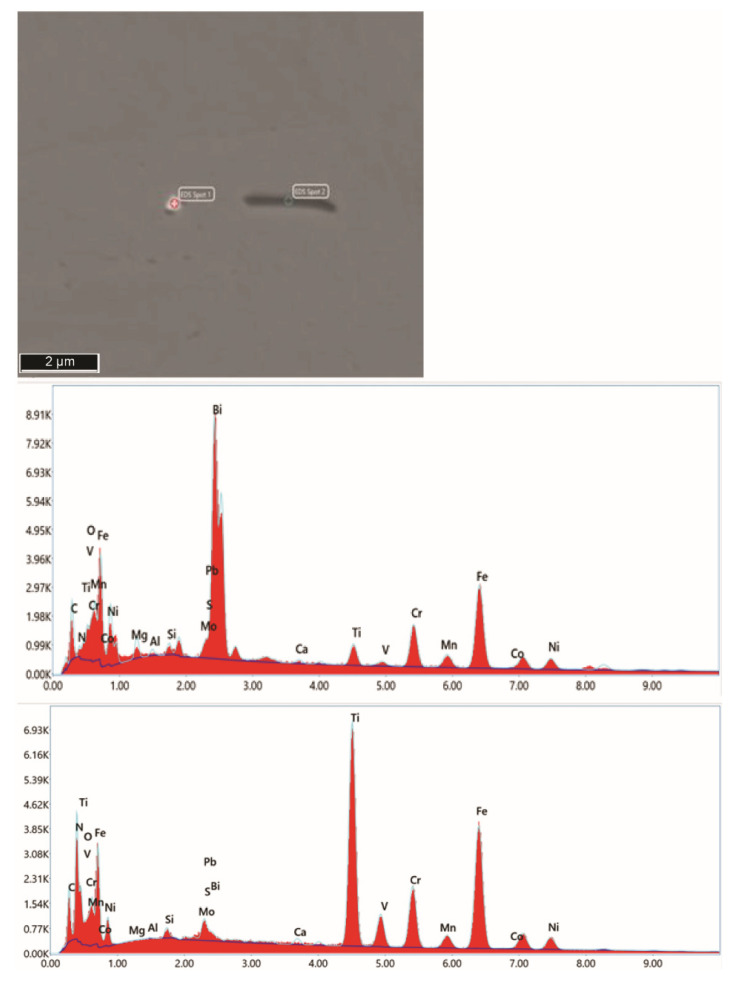
Bi and Pb enrichments in the hot-rolled plate of steel A (FE-SEM-EDS; mag. 10,000×).

Separate Bi and Pb enrichments were also found in the hot-rolled plate of steel A (Figure 13).

Based on the results (see Figure 2, Figure 10, Figure 11 and Figure 13), it is most plausible that a recycled FeTi-cored wire was the main source of the elevated levels of impurities detected in the form of cosegregations.

## 4. Discussion

During the production of Ti-stabilized Cr-Ni-Mo austenitic stainless steel, the steel is stabilized by titanium. In the production of the steels under investigation, a recycled ferrotitanium-cored wire (FeTi) was used.

In representative microstructures of the analysed hot-rolled steel plates A, B, and R, large accumulations of impurities and oxygen were observed. Moreover, it is obvious that not only Ti but large amounts of additional impurities were introduced into the steel by alloying using a recycled FeTi (see Ti, O, S, Mo, Bi and Pb maps in Figure 2). In addition, a considerable proportion of the volume was also represented by segregations of the impurity elements Mo, S, Pb, and Bi cosegregations (Figure 10 and Figure 11).

The quality requirements of the FeTi wire are its chemical composition, size, and cleanliness. The chemical compositions of the FeTi alloys are defined by the standard specifications for ferrotitanium, e.g., ASTM A324—08(2019), GOST 4760-91, etc. [22]. The tolerable levels of elements may vary. Usually, the Ti-Al-V tolerances are defined. In addition, the maximum contents of N, Pb, Bi, and Sn are very important [22,27].

The cumulative content of impurity elements in the chemically very inhomogeneous FeTi-cored wire was relatively high (Table 2), and the contents of Bi, Sn, Al, V, and Mo were especially high. In addition, an increased value of O in the FeTi wire was confirmed by microchemical analysis. The average values of selected elements in the FeTi wire are provided in Table 4. The proportion of insoluble Al was 0.18 wt. %. This could have acted as a source of additional O.

Based on the composition of the FeTi wire (see Table 2 and Table 4), it is very likely that Ti6Al4V scrap was used (high proportion of Al and V). Ti6Al4V is an (α + β) titanium alloy that is very commonly used in aviation [27]. The increased contents of O, C, S and Mo, however, were probably contaminants from the cutting process. MoS_2_ and graphite are often used as additives to lubricants in the cutting of Ti6Al4V and other titanium alloys. During the cutting process, MoS_2_ and graphite adhere to the Ti chips. This makes them very difficult to remove. It is also known that the chips oxidize during cutting.

In general, the Mo-S system is very important in extractive metallurgy [36]. Molybdenum disulphide (MoS_2_) is widely used as a dry lubricant because of its low friction and robustness. According to the relevant literature [37], the presence of Bi impurities in MoS_2_ is confirmed to be in the form of bismuth oxide or sulphate compounds (Bi_2_O_3_/Bi_2_O_5_/Bi_2_(SO_4_)_3_). Bi impurities were also detected in elevated levels in the cored FeTi wire.

The presence of low-melting-point eutectic phases can initiate liquation cracking [38]. The hot cracking of steels is dependent on the levels of impurity elements. The impurities S, P, Pb, Bi, Sn, and Sb affect the hot workability of stainless steels. S and Pb segregate to the phase and solidification grain boundaries where cracks appear during hot deformation [39]. Particularly in Ti-stabilized, fully austenitic stainless steels, various carbosulphides and eutectics in conjunction with S, N, and C are deleterious [40].

The melting point of pure Bi is 271.4 °C. In a steel melt, Bi has a large local vapour pressure [41]. Moreover, Bi is well known to form morphologically diverse Bi-sulphides [42,43]. Most Bi particles adhere to preexisting sulphides in steel. Consequently, high Bi content in steel results in an increase in the number, average area, and average diameter of large-sized sulphide inclusions [43]. It was also reported that in austenitic stainless steels, additions of (Bi+Cu+S) improve the steel’s machinability and tensile properties [44]. However, the hot workability of Bi-bearing steels seems to depend greatly on the size, volume fraction, type, and distribution of the inclusions [45]. Low-melting-point Bi segregations can also be formed at austenite grain boundaries at 950–1100 °C [46,47].

Furthermore, it was reported that in the ternary Bi–Mo–O system, the following compounds can be formed [48]:

- Bi_2_(SO_4_)_3_ with a melting point of 405 °C;
- Bi_2_O_3_ with a melting point of 817 °C;
- Bi_2_O_5_, usually stable only in combination with Bi_2_O_3_ and not alone;
- in the ternary Bi–Mo–O system, the existence of several bismuth molybdates Bi_x_Mo_y_O_z_ with melting points below 1000 °C is possible.

Steel production failures, in terms of inappropriate mechanical, chemical, thermal, or other properties of steel, are more or less regularly detected in ex post analyses.

However, a classification method based on a calculation of the Mahalanobis distance (MD) between the inclusions (i.e., nonmetallic inclusions and segregations) can be used to detect abnormalities. In this method, it is necessary to define all the deviations in terms of binarity (i.e., normal–abnormal).

Although many material properties exhibit a continuous span, the standards for specific steel grades determine the limit values and can thus also be considered as binary (normal vs. abnormal). On the other hand, many properties such as surface defects, cracks, etc. are inherently binary (normal vs. abnormal). The remaining production without detected defects or within the specified tolerances can be considered as the normal production class. Using thresholding of the MDs, we could use the MD as a predictive classification model, although with some precautions and limited predictive accuracy [35]. Nevertheless, the MD has found various applications [34]. MD is used to construct a continuous measurement scale to discriminate observations and measure the level of abnormality of abnormal observations that are compared to a group of normal observations [34].

The MD, as a measure of the difference between only two classes, offers some possibilities for the automatic detection of anomalies in steel measured indirectly using data obtained with the automatic detection of nonmetallic inclusions. Since the MD is by nature a comparative method, the data obtained (automatic detection of nonmetallic inclusions) on charges without any, or with noticeable, anomalies could be used for the normal or reference class.

As expected, the data collected using more charges of the same steel grade and the same production technology led to a more stable normal class. The MD calculation and threshold calculation can be automatic, generally without human intervention. To use the MD as an outlier detector, we need only determine an additional threshold in terms of, e.g., the percentage of outliers for additional data and could automatically take some action in terms of additional report generation (e.g., ternary plots and/or notification for human supervision). The application of MDs in this case leads to an abundance of MDs obtained from a single sample (or charge in this case), since an MD is obtained for each nonmetallic inclusion. The abundance of data in the distribution space offers an excellent and diverse opportunity for differentiation. Perhaps the most obvious drawback of MDs is that they do not give insight into the metallurgical processes and chemistry involved in the formation of nonmetallic inclusions, which is contrary to established ternary plots. As shown in the paper (see Figure 8 and Figure A1 and Figure A2 in Appendix A) the application of an MD combined with ternary plots extends the NMI data-analysis possibilities. Although the use of MD on NMI features for two-class separation (normal vs. abnormal) and NMI features for clustering according to their types stem from same data, their purposes and uses are very different.

## 5. Conclusions

Selected samples of three charges of scrap-based, Ti-stabilized, Cr-Ni-Mo, austenitic stainless steels in the form of hot-rolled steel plates were characterized. Based on automated metallographic analyses of the inclusions in representative microstructures (i.e., nonmetallic inclusions and segregations) a high-quality characterization in terms of steel cleanliness of the hot-rolled plates was possible. In addition to this, the classification of materials based on a calculation of the Mahalanobis distance (MD) between inclusions was possible.

The following conclusions can be drawn:

- The recycled FeTi-cored wire was the main source of elevated levels of impurities detected in the hot-rolled, Ti-stabilized stainless-steel plates.
- Insoluble impurities that are present in the FeTi wire for alloying of Cr-Ni-Mo stainless steel can greatly affect the final quality of the hot-rolled plates.
- Because of a high oxygen content in the used FeTi wire, the elemental Al could react with Ti_x_O_y_ to form complex, nonmetallic inclusions that were very stable at the fabrication temperature of the steel before continuous casting.
- The occurrence of Mo, S, Pb, and Bi segregations was associated with the use of the recycled FeTi wire and the use of MoS_2_ in the mechanical preparation of the FeTi.
- In calculations of MDs, the steel charge with the smallest number of nonmetallic inclusions and segregations was set as a reference class.
- MDs, as a measure of the difference between only two classes, offer some possibilities for the automatic detection of abnormalities in steel measured indirectly using the data obtained with the automatic detection of inclusions.
- However, a combination of metallographic and mathematical techniques is recommended.
- The selection of outlier inclusions based on their MDs and their back-representation into ternary diagrams gave relevant metallurgical information about the abnormalities.
- The advantage of this technique is that the calculations of the MD and the threshold can be fully automated.

## Figures and Tables

**Table 1:** Characteristics of Ti-stabilized Cr-Ni-Mo steel plates under investigation.

Chemical Composition/in wt. %											
Sample	Cr	Ni	Mo	Ti	C	O	N	S	Pb	Bi	Fe
A	17.1	10.7	2.1	0.4	0.041	0.0026	0.0104	<0.001	<0.01	<0.01	Bal.
B	17.1	10.7	2.1	0.3	0.024	0.0059	0.0104	<0.001	<0.01	<0.01	Bal.
R	17.0	11.9	2.1	0.3	0.049	0.0013	0.0080	<0.001	<0.01	<0.01	Bal.
Tolerance wt. %	±0.2	±0.2	±0.05	±0.001	±0.003	±0.00002	±0.0005	±0.0005	±0.005	±0.005	/
**Charpy V-notch impact toughness/in J**											
Sample	25 °C			−45 °C			−196 °C				
A	216.5 ± 0.5			219.0 ± 1.0			178.0 ± 6.0				
B	264.0 ± 0.0			266.0 ± 0.0			212.0 ± 2.0				
R	185.5 ± 0.5			163.5 ± 0.5			136.0 ± 2.0				
				**Vickers hardness (HV10)**							
A				152 ± 4							
B				150 ± 12							
R				147 ± 1							

Differences in the mechanical properties of the steel plates were attributed to the differences in their chemical compositions.

**Table 2:** Chemical compositions of the randomly selected samples of FeTi-cored wire (in wt. %).

Element	Sample 1	Sample 2	Sample 3	Tolerance/wt. %
Al	2.7	3.1	3.2	±0.001
Bi	0.10	0.08	0.13	±0.005
Cr	0.38	0.35	0.34	±0.2
Fe	17.1	16.1	15.0	±0.2
Mn	0.10	0.14	0.09	±0.05
Mo	0.33	0.67	0.44	±0.05
Ni	0.42	0.55	0.39	±0.2
Pb	<0.01	<0.01	<0.01	±0.005
Sb	<0.01	<0.01	<0.01	±0.005
Sn	0.21	0.24	0.34	±0.005
V	1.9	1.7	2.0	±0.01
Zr	0.22	0.25	0.36	±0.005
Si	0.29	0.29	0.30	±0.02
C	0.17	0.16	0.17	±0.003
S	0.009	0.010	0.010	±0.0005
N	1.03	0.75	0.77	±0.005
O	2.02	1.47	2.06	±0.0005
Ti	Bal.	Bal.	Bal.	—

**Table 3:** Number of detected inclusions in samples A and B and reference sample R and the size class, class frequency, and average size of these inclusions.

	Class Frequency		
Size Class	Sample A	Sample B	Sample R
**1** < 0.015 µm^2^	3824	5490	3943
0.015 < **2** < 0.02 µm^2^	1400	1607	1276
0.02 < **3** < 0.03 µm^2^	3043	3361	2259
0.03 < **4** < 0.04 µm^2^	1411	1393	839
0.04 < **5** < 0.05 µm^2^	1506	1471	799
0.05 < **6** < 0.1 µm^2^	2705	2425	1176
0.1 < **7** < 200 µm^2^	1969	958	1049
Inclusions in total:	15,858	16,705	11,341
Average size of inclusions/µm^2^:	0.20 ± 2.36	0.13 ± 1.46	0.25 ± 2.54

**Table 4:** Average values of the selected elements in the recycled FeTi-cored wire (wt. %).

% C	% S	% Al—Soluble	% Al—Insoluble	% Si
0.16 ± 0.01	0.01 ± 0.001	3.1 ± 0.2	0.18 ± 0.03	0.29 ± 0.02

## Data Availability

The data used to support the findings of this study are available upon request.

## Abbreviations

Besides chemical formulae of compounds, the following abbreviations are used in the manuscript:

AISI	American Iron and Steel Institute
AOD	Argon oxygen decarburization
ASTM	American Society for Testing and Materials
BSE	Back-scattered electrons
BSI	Back-scattered electron imaging
CE	Circular economy
EAF	Electric arc furnace
ECD	Equivalent circular diameter
EDS	Energy-dispersive spectroscopy
EOL	End-of-life
FE-SEM	Field emission scanning electron microscopy
GOST	Gosudarstvennii Obscesoiuznii Standard (Russian)
ICP-OES	Optical emission spectrometer with inductively coupled plasma
ISO	The International Organization for Standardization
LF	Ladle furnace
LN2	Liquid nitrogen
MD	Mahalanobis distance
NMI	Nonmetallic inclusion
OM	Optical microscopy
VOD	Vacuum oxygen decarburization
Z	Atomic number of a chemical element
